# Minimally invasive esophagectomy via Sweet approach in combination with cervical mediastinoscopy for esophageal squamous cell carcinoma: a case series

**DOI:** 10.1097/IJ9.0000000000000045

**Published:** 2017-11-08

**Authors:** Wenxiang Wang, Baihua Zhang, Xu Li, Jie Wu, Zhining Wu, Yan Ding, Desong Yang, Jinming Tang, Min Su, Junliang Ma, Xianman You, Jianping Liang, Yong Zhou

**Affiliations:** The 2nd Department of Thoracic Surgery, Hunan Cancer Hospital, The Affiliated Cancer Hospital of Xiangya School of Medicine, Central South University, Changsha, Hunan Province, China

**Keywords:** Esophageal cancer, Minimally invasive esophagectomy, Sweet approach, Mediastinoscopy

## Abstract

**Objective:**

Minimally invasive esophagectomy (MIE) is increasingly used for the treatment of esophageal cancer. However, MIE via the Sweet approach has seldom been reported owing to the challenging procedure for a mediastinal lymph node. Thus, the approach of MIE via left-sided thoracoscopy coupled with video-assisted cervical mediastinoscopy (MIE-SM) was explored for eradicating the mediastinal lymph nodes and recurrent laryngeal nerve; the incidence of perioperative complications, mortality, and surgical radicality were analyzed.

**Materials and Methods:**

Thirty patients with esophageal carcinoma underwent MIE-SM between June 2014 and February 2016. The primary outcome was postoperative morbidity within 2 weeks postsurgery. The secondary outcome was surgical radicality, including the circumferential margins, and the number of lymph nodes dissected.

**Results:**

The MIE-SM was completed in all patients within 367.6±68.7 minutes. The incidences of postoperative morbidities including pulmonary complications, anastomotic leakage, chylothorax, or recurrent nerve injury were 43.3%.

**Conclusion:**

The MIE-SM was utilized for the first time to reduce the disadvantage of purely Sweet and McKeown approach, with favorable efficacy in the mediastinal and laryngeal recurrent nerve lymph node eradication. Thus, MIE-SM might be a promising alternative approach in treating esophageal cancer in selected patients.

Esophageal cancer has an extremely poor prognosis worldwide. A total of 477,900 patients were newly diagnosed with esophageal cancer in China in 2015, accounting for more than half of those worldwide, and an estimated 375,000 patients died of this disease^1^. Although surgical resection remains the primary curative option for resectable esophageal cancer, these approaches and lymph node dissection are yet controversial. The Sweet esophagectomy (left posterolateral thoracotomy) is not preferable in China due to inadequate lymph node dissection in the superior mediastinum; however, it is still widely performed in patients with cancer localized in the middle or lower third of the thoracic esophagus. In contrast, Ivor-Lewis esophagectomy (right-sided thoracotomy) offers superior visualization of the upper mediastinum and allows extended lymphadenectomy; nevertheless, it is performed less frequently owing to a frequent association with high postoperative morbidity, excessive blood loss, and prolonged surgical duration and hospital stay^2^,^3^. Thus, the Sweet procedure continues as an option for the treatment of middle-third and lower-third esophageal cancers.

Several recent studies have focused on minimally invasive esophagectomy (MIE) in order to reduce surgical trauma and morbidity. In comparison to open procedures, minimally invasive Ivor-Lewis or McKeown esophagectomy may allow better visualization of the mediastinum and extensive thoracic and abdominal lymphadenectomy^4^–^7^. With respect to perioperative complications, the minimally invasive approaches can also reduce the morbidity of pulmonary complications, length of intensive care unit (ICU) and hospital stay, and rate of recurrent laryngeal nerve injury^8^,^9^. In addition, MIE can achieve long-term survival rates similar to those of open surgery^7^. In elderly patients, MIE can provide a long disease-specific survival time^2^. As a result, the minimally invasive approaches are being frequently used and considered as suitable alternatives to open esophagectomy.

To the best of our knowledge, currently, MIE is performed via right-sided thoracoscopy owing to better visualization of the thoracic esophagus. Few reports have described MIE via the left-sided (Sweet) approach. In an attempt to understand the feasibility and safety of the minimally invasive Sweet approach, for the first time, we performed MIE via left-sided thoracoscopy in several patients and achieved favorable short-term results. Considering the limitations of this approach with respect to the exposure of the upper mediastinum and extent of lymph node dissection, we used video-assisted mediastinoscopy via the neck to improve lymphadenectomy on complementation. The present study performed MIE via the Sweet approach coupled with cervical mediastinoscopy (MIE-SM) in patients with esophageal squamous cell cancer that was localized in the middle and lower third of the thoracic esophagus at a high-volume cancer center. We also assessed the incidence of perioperative complications, mortality, and surgical radicality.

## Materials and methods

Ethical approval for this study work was reviewed and approved by the Ethics Committee of Hunan Cancer Hospital, Changsha, China. The data of 30 patients who underwent MIE-SM at the 2nd Department of Thoracic Surgery, Hunan Cancer Hospital of Xiangya School Medicine (Changsha, Hunan Province, China) from June 2014 to February 2016 were assimilated. Written informed consent was obtained from all participants at the beginning of the study. Surgery was performed by experienced thoracic surgeons.

Inclusion criteria: patients who presented esophageal cancer located in the middle and lower third of the thoracic esophagus resectable disease (cT1-3, N0-1, M0), no evidence of distant metastasis, no enlarged lymph nodes in the upper mediastinal, cervical, or celiac areas, availability of stomach for use as a conduit, and histologically confirmed squamous cell cancer were included in the study. The exclusion criteria were enlarged lymph nodes in the upper mediastinum (>5 mm), esophageal cancer located in the upper third of the thoracic esophagus, history of esophageal or gastric surgery, presence of neoadjuvant therapy, age older than 75 years, and severe major organ dysfunction or other diseases that prevented the performance of minimally invasive surgery.

The primary outcome was postoperative morbidity, defined as the incidence of pulmonary infection, pulmonary atelectasis, recurrent laryngeal nerve injury, postoperative hemorrhage, hiatal herniation, anastomotic leakage, chylothorax, pyothorax, wound infection, or reoperation for any reason within 2 weeks postsurgery. The secondary outcome was surgical radicality, including the circumferential margins and number of lymph nodes dissected, length of hospital and ICU stays, intraoperative data, such as the operating time (min) calculated from skin incision to skin closure, estimated blood loss (mL), and conversion from thoracoscopy or laparoscopy to an open procedure, as well as, 30-day postoperative mortality, defined as death due to any cause after surgery.

The data are expressed as mean±SD or median (range) for continuous variables as appropriate. The distributions of dichotomous data are expressed in percentages.

## Results

### Patients’ characteristics

From June 2014 to February 2016, 30 patients with esophageal carcinoma who underwent MIE-SM at the Hunan Cancer Hospital were enrolled. The patients’ demographic and clinicopathological characteristics were analyzed in **Table 1**.

**Table 1:**
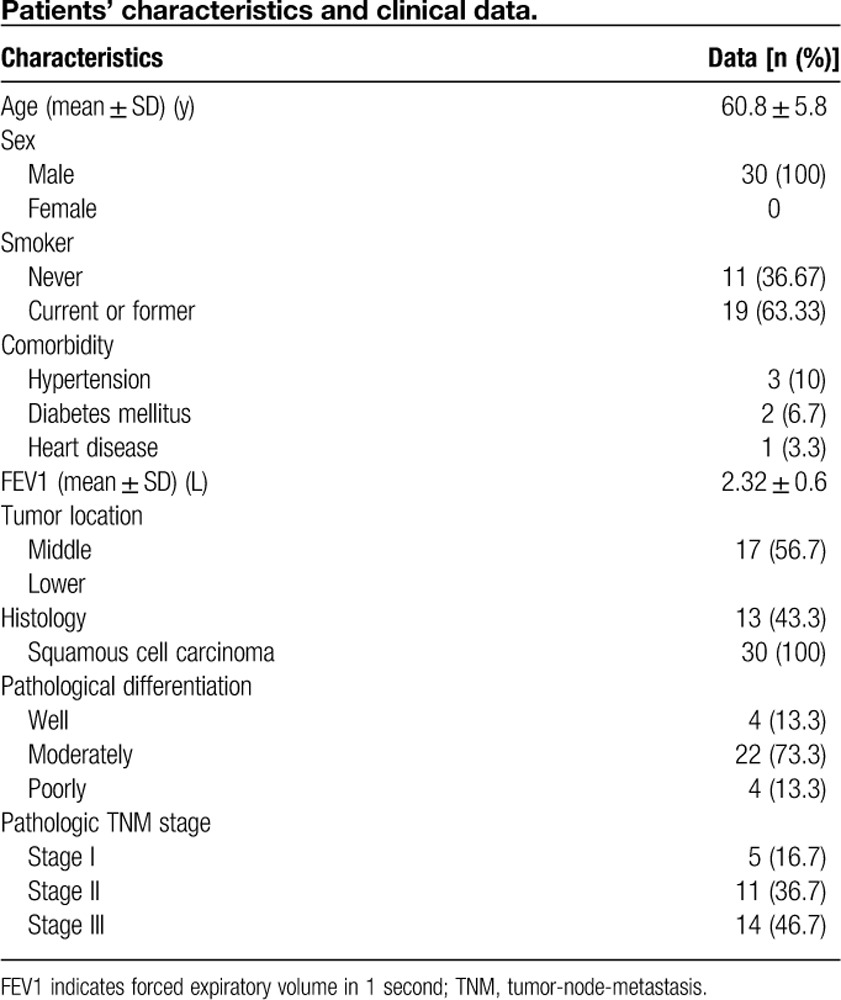
Patients’ characteristics and clinical data.

### Procedure

**Figure 1:**
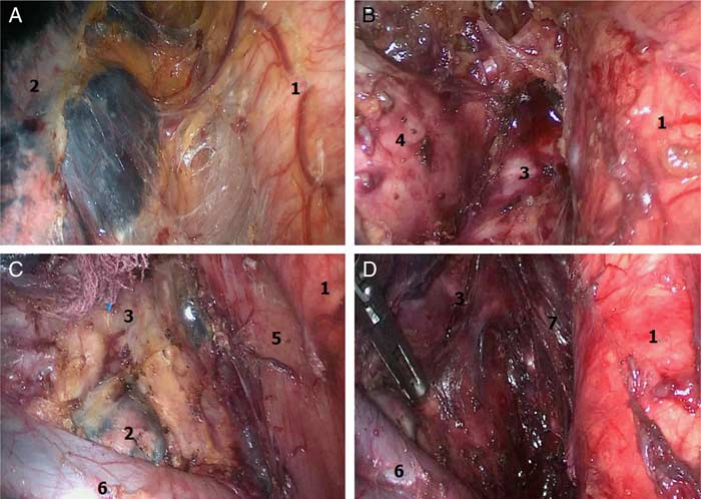
Thoracoscope-based intrathoracic operation (A, separate thoracic segment of the esophagus; B, dissect lymph node near the lower trachea and left main bronchus; C, dissect lymph node near the middle esophagus and separate the esophagus; D, dissect subcarinal lymph node and separate esophagus. 1, Thoracic aorta; 2, left lower lung; 3, left bronchial artery; 4, left pulmonary artery; 5, thoracic esophageal; 6, left lower pulmonary vein; 7, right bronchial artery).

**Figure 2:**
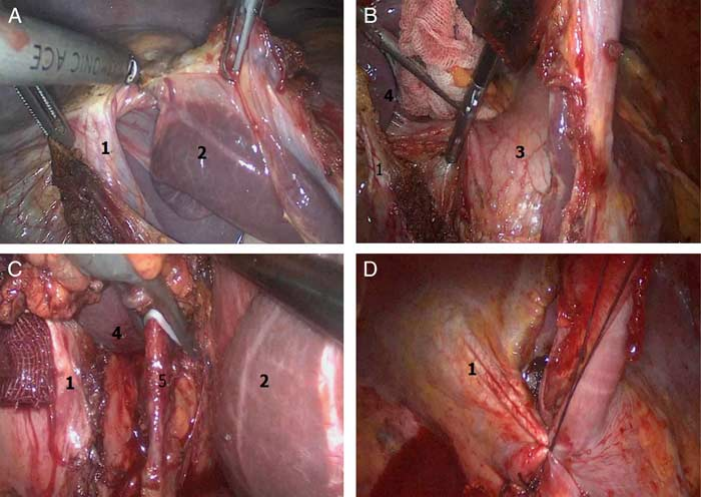
Cavity mirror internal operations (A, divide diaphragm; B, resection of short gastric vessels and separate lesser curvature of the stomach; C, HEMLOCK treatment of left gastric artery; D, interrupt and suture the diaphragm. 1, Diaphragm; 2, liver; 3, stomach; 4, spleen; 5, left gastric artery).

**Figure 3:**
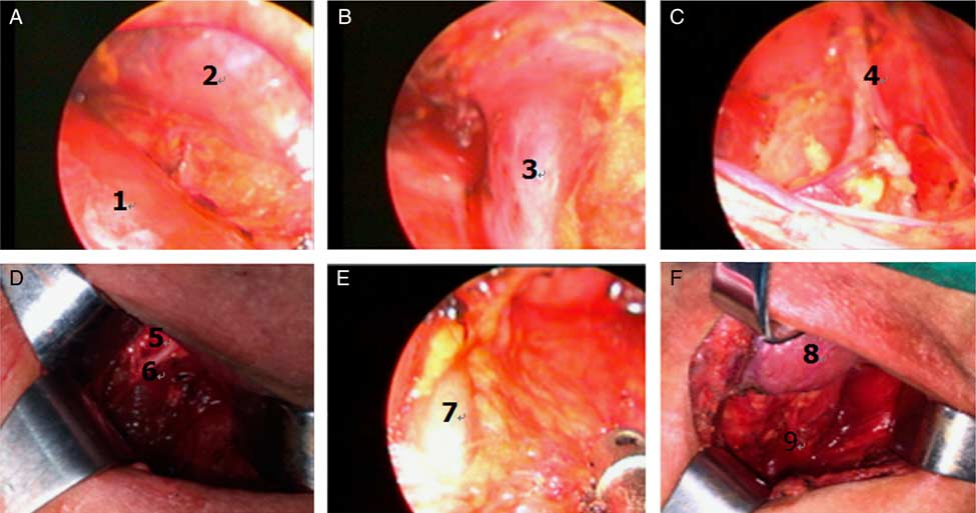
Lymph node dissection of bilateral recurrent laryngeal nerve chain by cervical mediastinoscopy (1, trachea; 2, superior vena cava; 3, umbilical vein; 4, thoracic inner segment of right recurrent laryngeal nerve; 5, right thyroid; 6, neck segment of right recurrent laryngeal nerve; 7, thoracic inner segment of left recurrent laryngeal nerve; 8, right thyroid; 9, neck segment of left recurrent laryngeal nerve).

1. The 4-port method was utilized in chest operation. The patients were placed in the right lateral position. The first observation port for the thoracic operation was created ∼1 cm at the ninth intercostal axillary line. The main operating port was located at the seventh intercostal axillary line followed by creating auxiliary operation ports ∼1 cm on the fifth intercostal axillary line and eighth midaxillary line for traction of the lungs and exposure to esophagus, of which, the fifth intercostal axillary midline incision was used as the second observation hole in the follow-up operation. Chest and abdominal cavity operation were performed under auxiliary artificial pneumothorax with 8–10 mm Hg pressure.
  After dissection of the esophageal triangle, the thoracic aortic mediastinal pleura was dissected along the longitudinal axis of the esophagus (**Fig. 1A**). The esophagus was separated up to the aortic arch level using a harmonic scalpel. The lymph nodes near the para esophagus, lower trachea, and the left main bronchia were removed (**Fig. 1B**). Subsequently, the pulmonary mediastinal pleura was dissected along the longitudinal axis of the esophagus downstream to the diaphragm hole to separate the esophagus. The lymph nodes next to the esophagus, lower pulmonary vein, pulmonary ligament, and phrenic were removed (**Fig. 1C**). The subcarinal lymph node was dissected together with the surrounding adipose tissue, and the esophagus was isolated to the level of aortic arch (**Fig. 1D**).
  After placing the thoracoscope into the second observation port into the patient in a supine position at 30 degrees, the diaphragm was resected along the hiatal esophagus (**Fig. 2A**) and the right diaphragm in the chest wall. After transection of the liver and stomach ligament, the lesser omentum was dissected, followed by separation of the ligament between spleen and stomach and stomach small vessels. Then, the stomach curvature was isolated (**Fig. 2B**). The stomach was elevated, and the left gastric blood vessels were exposed from the small curved side. The left gastric vein with a harmonic scalpel was dissected, and the left gastric artery was treated using HEMLOCK (**Fig. 2C**), followed by dissection of the surrounding lymph nodes and continual bending along the stomach size from the stomach until the complete release of the pylorus. Consequently, the right aneurysm of the retina and the right gastric artery were protected and the lymph nodes near the lateral stomach curvature were dissected.
  Then, the main operating hole was extended to ∼4 cm, and the stomach of the body was pulled out. A tube-type stomach was fashioned using the straight line cutting instrument, stitching, and embedding the edge of the suture. The tumor segment was removed from the esophageal traction at the bottom of the heart line, the sutures interrupted, and the diaphragm closed (**Fig. 2D**). After placing a thoracic drainage tube, the chest incision was stitched.
2. The patient was then repositioned to the supine position. An incision, ∼5 cm, along the skin pattern was located on the sternum 2 cm. After dissection of the skin, subcutaneous tissue, and fascia, the mediastinoscope was placed in the thoracic cavity from the right side of the trachea. The lymph nodes in the superior vena cava—tracheal space between the nameless arterial and odd vein—were dissected (**Figs. 3A, B**). The mediastinoscopy was used appropriately to tap the nonspecific artery, separate the right recurrent laryngeal nerve back to the fold, and dissect the lymph nodes. The mediastinoscopy vision was utilized for isolating the laryngeal nerve in the thoracic cavity and dissecting the surrounding lymph nodes (**Fig. 3C**).
  Then, the right side of the retraction was pulled back to the neck sheath and trachea, separating the right recurrent laryngeal nerve and dissecting the lymph nodes surrounding the right side of the neck to the recurrent laryngeal nerve chain up to the level of thyroid (**Fig. 3D**). The same method was used to isolate the left side of the recurrent laryngeal nerve to the left main bronchus, followed by the dissection of the surrounding lymph nodes (**Fig. 3E**). After pulling away from the left carotid sheath and trachea by the retractor, the left laryngeal recurrent nerve cervical segment to the thyroid levels was isolated, and the neck lymph nodes proximal to the left recurrent laryngeal nerve was dissected (**Fig. 3F**).
  Subsequently, the mediastinoscope was placed from the left trachea. After dissection of the tracheal-esophageal fascia, the thoracic and cervical esophagus were isolated from the left main bronchus downstream to the left level of thyroid. After elevating the tube to the neck, the esophageal-gastric mechanical anastomosis was performed on the left side of the thyroid level. The nasogastric decompression tube and nutritional support branch were placed. Finally, the neck drain tube was placed, and the cervical incision was sutured.
3. After surgery, each patient was transferred to the ICU for stabilization and extubation, and then to the general surgical ward on the following day. Patient-controlled analgesia with intravenous opioids was administered during the first 3 days postsurgery, and the patients were encouraged to move out of bed. Moreover, the patients were fed through a nasoenteral tube on postoperative day 1. On postoperative days 4–5, oral feeding was started, and enteral feeding was decreased. Consequently, the patients were discharged when they could ingest semisolid food and oral analgesia. The first follow-up was conducted 4 weeks postsurgery.

### Morbidity and mortality

One patient who underwent the MIE-SM operation was converted to open surgery because of intraoperative injury to the splenic artery; all other patients underwent successful MIE. None of the patients died of postoperative complications within 30 days after surgery. The incidences of pulmonary infection/atelectasis (20.0%), chylothorax (6.7%), anastomotic leakage (3.3%), and recurrent laryngeal nerve injury (13.3%) were summarized. Moreover, the operating time was 367.6±68.7 minutes, the length of ICU stay was 40.2±63.8 hours, and the length of hospital stay was 12.5±4.2 days. The mean intraoperative blood loss volume was 320.0±138.0 mL. One patient underwent a reoperation by thoracotomy to control chylothorax.

### Lymphadenectomy

Radical resection (R0) was achieved in all patients. The number of dissected lymph nodes was 25.7±10.1. In the recurrent laryngeal nerve regions, 4.1±2.7 lymph nodes were retrieved. The average number of lymph nodes retrieved in the upper mediastinum was 8.7±5.9, while that in the middle/lower mediastinum was 10.2±8.8. The lymph node metastasis was detected in 15 patients (50%).

## Discussion

As open transthoracic esophagectomy has a high incidence of complications, especially for traditionally open Ivor-Lewis and McKeown esophagectomy^10^–^12^, increasing attention has been focused on minimally invasive surgery. Thoracoscopy coupled with laparoscopic operation-based Ivor-Lewis or McKeown (left cervical, right chest, and median abdominal) surgery is the primary curative option for MIE. However, these 2 procedures present several limitations, such as multiple surgery incisions, surgical steps cumbersome, and a prolonged operation time. Moreover, the thoracic doctors need to learn laparoscopic technology specifically, which might be slightly difficult for some surgeons, and the learning curve is relatively long. Specifically, the technology requires conventional stapler or oral delivery anvil head (such as Orvil system) for intrathoracic anastomosis according to the Ivor-Lewis method. The operation, which is difficult and expensive, is not conducive to the promotion and application.

In contrast, the left thoracic incision (Sweet) can obtain a good curative effect in the middle and lower segment of the early esophageal cancer. The long-term effect is not inferior to the right thoracic incision. However, the deficiency of upper mediastinum and bilateral recurrent laryngeal nerves on lymphadenectomy limit the application of Sweet surgery. The video-assisted mediastinoscopy technique demonstrates the mediastinal anatomical structure and resection of the superior mediastinal lymph nodes, especially the bilateral laryngeal nerve chain lymph node dissection. It is an excellent complement and adequate for the left thoracic pathophyal radical resection of esophageal cancer.

Therefore, we attempted to resect esophageal cancer as well as gastric dissociation under the left thoracic approach. In addition, the combination of mediastinoscopy for lymph nodes dissection in the upper mediastinum and along the bilateral recurrent laryngeal nerves was performed. We constantly improved and optimized the specification for this operation, from the initial open thoracic surgery to the thoracoscope-assisted small incision surgery, and finally to thoracoscopic surgery. In the present study, we performed MIE-SM esophagectomy in 30 consecutive patients, and none of the patients died of perioperative complications within 30 days after surgery; 1 patient was converted to open surgery because of hemorrhage. The average operating time of MIE-SM (367.6±68.7 min) similar to that of MIE-MC reported in previous studies (349.9±86.3 min). The incidence of postoperative morbidity in the MIE-SM operation (43.3%) did not alter significantly from that of MIE-MC reported in previous studies (42.9%–49.5%)^13^,^14^. As a result, we speculate that MIE-SM esophagectomy is surgically safe and does not increase the risk of the operation. The work was reported in line with the PROCESS criteria of Preferred Reporting of Case Series in Surgery^15^.

The surgical incision is essential for the thoracoscopic resection of esophageal carcinoma. In the present study, we selected left thoracic incision according to our experience and the design of the right thoracoscopic incision by Luketich^13^. The first port in the ninth intercostal axillary line can fully expose the thoracic esophagus below the aortic arch, which allows the surgeon to dissociate the esophagus and dissect the lymph nodes under direct visualization. Moreover, the pulmonary interference can be reduced with positive pneumothorax.

The normal esophagus was dissociated in the upper and lower ends of the tumor and treated with an ultrasonic knife along the anatomic planes after traction. The safe and effective hemostasis keeps the field of vision clean. The exposed field of vision is not inferior to the right side of the thoracoscope. One of the difficulties of the left thoracoscopic surgery is the dissociation of esophagus behind and above the aortic arch. The thoracoscope was extended to the aortic arch for observation, and the surgeons could dissociate the esophagus under direct visualization. In addition, the azygos vein arch and the lower trachea can achieve adequate exposure, and the tracheal lymph nodes can be removed simultaneously. The thoracoscopy was then transferred to the second port for observation. The pressure could be increased to 10–14 mm Hg after opening the diaphragm, and the abdominal cavity exposed fully with artificial pneumoperitoneum. Similar to the procedure of the left thoracotomy, it is not difficult to dissociate the proximal stomach.

The current study has revealed several advantages of MIE-SM over the conventional open procedure. One of the advantages of this operation is to avoid multiple abdominal wall incision and reduce the psychological impact on patients. The mobilization of the thoracic esophagus and stomach can be achieved via left-sided video-assisted thoracoscopy. As a minimally invasive technique, MIE-SM causes less injury and pain and avoids the laparoscopic incisions that are required by the minimally invasive Ivor-Lewis and McKeown approaches. The minimally invasive surgical techniques aims to reproduce the radicality of open operations while achieving similar oncologic efficacy. In this study, all patients achieved radical resection (R0). However, radical lymphadenectomy is a critical aspect of the surgical treatment of esophageal cancer. Two-field or 3-field lymph node dissection is beneficial in some patients and balances the extent of lymphadenectomy with the morbidity of the operation^16^,^17^. The number of lymph nodes resected in the MIE-SM was 25.7±10.1, which was higher than that of other operations, such as MIE-MC (18.8±8.0)^13^,^18^, suggesting that lymphadenectomy under left-sided thoracoscopy is surgically applicable.

The present study indicated that patients might benefit from MIE-SM esophagectomy; however, a prospective randomized clinical trial comparing the short-term morbidity and long-term outcomes between MIE-SM and other operation is essential. Further studies including an additional number of patients should be performed to optimize the technical procedure and further evaluate the perioperative safety and long-term oncological outcomes of MIE-SM.

## Conclusion

In conclusion, MIE-SM appears to be safe for patients with mid and lower thoracic esophageal cancer. MIE-SM can conveniently harvest the lymph nodes in the upper mediastinal and bilateral recurrent laryngeal nerve regions. Therefore, MIE-SM should be considered as a promising alternative approach for the treatment of esophageal cancer in selected patients.

## Ethical approval

Ethical approval for this study work was reviewed and approved by the Ethics Committee of Hunan Cancer Hospital, Changsha, China.

## Sources of funding

Supported by the grants from Health and Family Planning Commission of Hunan Province of China (B2015-113).

## Author contribution

W.W.: design, surgical operation, data collection, analysis, writing. B.Z. and X.L.: surgical operation, data collection, analysis, writing. J.W., Z.W., Y.D., D.Y., J.T., J.L., Y.Z.: surgical operation, data collection. M.S., J.M., X.Y.: analysis, writing.

## Conflict of interest disclosure

The authors declare that they have no financial conflict of interest with regard to the content of this report.

## Research registration unique identifying number (UIN)

Researchregistry2942.
